# Resistance to pyrethroid and organophosphate insecticides, and the geographical distribution and polymorphisms of target-site mutations in voltage-gated sodium channel and acetylcholinesterase 1 genes in *Anopheles sinensis* populations in Shanghai, China

**DOI:** 10.1186/s13071-019-3657-7

**Published:** 2019-08-09

**Authors:** Yuan Fang, Wen-Qi Shi, Jia-Tong Wu, Yuan-Yuan Li, Jing-Bo Xue, Yi Zhang

**Affiliations:** National Institute of Parasitic Diseases, Chinese Center for Disease Control and Prevention, Chinese Center for Tropical Diseases Research, WHO Collaborating Centre for Tropical Diseases, National Center for International Research on Tropical Diseases, Ministry of Science and Technology, Key Laboratory of Parasite and Vector Biology, Ministry of Health, Shanghai, 20025 China

**Keywords:** Acetylcholinesterase 1, Insecticide bioassay, Knockdown resistance, Malaria, Organophosphate, Pyrethroid

## Abstract

**Background:**

In the final phase of China’s national programme to eliminate malaria by 2020, it is vitally important to monitor the resistance of malaria vectors for developing effective vector control strategies. In 2017 Shanghai declared that it had eliminated malaria; however, the insecticide resistance status of the primary malaria vector *Anopheles sinensis* remains unknown.

**Methods:**

We examined the pyrethroid and organophosphate resistance of *An. sinensis via* a bioassay of two populations from the Chongming District of Shanghai. The voltage-gated sodium channel (*VGSC*) and acetylcholinesterase 1 (*ace-1*) genes were partially sequenced to examine the association between resistance phenotype and target site genotype. In addition, the geographical distribution, polymorphism and genotype frequencies of insecticide resistance genes were examined using samples collected during routine mosquito surveillance in 2016 and 2017 from Chongming, Songjiang, Jiading and Qingpu Districts.

**Results:**

In Chongming District, the *An. sinensis* population near Dongtan National Nature Reserve showed resistance to pyrethroids, sensitivity to organophosphate, no knockdown resistance (*kdr*) mutations in the *VGSC* gene, and a low frequency (1.71%) of the *ace-1* 119S allele. An *An. sinensis* population near the Chongming central area (CM-Xinhe population) showed high resistance to pyrethroids and organophosphates and high frequencies of *kdr* 1014F and 1014C (80.73%) and *ace-1* 119S (85.98%) alleles. A significant association was detected between the homozygous *kdr* mutation 1014F/1014F and pyrethroid resistance in the CM-Xinhe population, indicating that the *kdr* mutation is probably recessive. Eight *kdr* genotypes with 1014F and 1014C substitutions were detected in the four surveyed districts of Shanghai. TTT and GGC/AGC were the dominant *kdr* allele and *ace-1* genotype, respectively, and were prevalent in most Shanghai *An. sinensis* populations.

**Conclusions:**

On the basis of our assessment of insecticide resistance gene mutations in Shanghai, we identified a *kdr* mutation-free population in Chongming Dongtan. However, high frequencies of target-site mutations of insecticide resistance genes were observed in most areas of Shanghai. Bioassays of *An. sinensis* populations in the central Chongming District indicated the high insecticide resistance status of *An. sinensis* populations in Shanghai. We accordingly recommend a restriction on insecticide usage and development of effective integrated pest/vector management interventions to support disease control efforts.

## Background

Malaria was once a major public health issue in China, and in the early 1970s approximately 24 million cases were reported in 24 malaria-endemic provinces [1]. The implementation of the Action Plan of China Malaria Elimination 2010–2020 launched in July 2010 has, however, substantially alleviated the malaria burden. In 2017, for the first time, no indigenous malaria cases were reported in China [2]. Thus, as planned, China is on track to eliminate malaria by 2020. The National Health Planning Commission confirmed that, at the provincial level, Shanghai was the first to achieve the goal of malaria elimination in 2017 [3]. However, this region faces the risk of malaria retransmission *via* imported cases. According to recent official annual reports on the national malaria status, even though there has been a precipitous decline in the number of indigenous cases, the number of imported malaria cases continued to rise in 2016 and 2017, as a consequence of increasing population movement, international business and tourist travel [4]. Control and prevention of the resurgence of malaria induced by imported cases have thus become major challenges in the post-control and post-elimination phases of malaria eradication [5]. Notably, populations of transmission vectors persist in areas in which malaria was previously endemic and continue to pose a threat of disease re-emergence. In China, four anopheline mosquito species have been reported to be important malaria vectors, namely *Anopheles sinensis*, *An. lesteri*, *An. dirus* and *An. minimus* [6]. Among these, distributions of the latter three species tend to be are very limited, with recorded population densities of < 0.001 light trap^−1^ h^−1^, according to the National Surveillance of Mosquitoes in China (2006–2015) [7]. In contrast, *An. sinensis*, the dominant *Anopheles* species in most regions of China, occurs at high population densities that peak from June to August [7, 8]. Vector control is acknowledged to be an effective measure for preventing malaria transmission, and has played an essential role in the prevention of both epidemics caused by indigenous cases and local secondary infections arising from imported cases [9]. However, an increase in the insecticide resistance of *Anopheles* populations, which can probably be attributed to the long-term overuse of insecticides in both agriculture and public health, represents a growing challenge for vector control [10, 11].

Pyrethroids, organophosphates, carbamates and organochlorines are four classes of insecticides recommended by the World Health Organization (WHO) for indoor residual spraying [12]. Pyrethroids have the advantages of high efficacy against mosquito vectors, low mammalian toxicity and short residual action. Consequently, pyrethroids are the only insecticides approved for use on long-duration insecticidal nets. As a consequence of long-term and large-scale deployment of insecticides for agricultural and public health protection, insecticide resistance was first detected in China in the 1990s, and has subsequently been identified in vector mosquitoes in most regions of China [10, 13]. Moreover, resistance to multiple classes of insecticides has become widespread, owing to the vector and pest control strategies used over the past half century, which possess considerable challenges for the effective control of vector-borne diseases.

The two main mechanisms of insecticide resistance involve, respectively, modification of the insecticide target site to reduce the affinity between the agent and the binding site and the upregulation of insecticide-detoxifying enzymes [14]. The voltage-gated sodium channel (*VGSC*) is the target site for pyrethroids and organochlorines. Mutation of codon 1014 of the *VGSC* gene has been linked to knockdown resistance (*kdr*) in several insects [15]. Similarly, mutation at codon 119 of the acetylcholinesterase gene 1 (*ace-1*) results in the single amino acid substitution of Gly to Ser and causes cross-resistance to carbamates and organophosphates [16]. Systematic literature reviews have revealed that the *kdr* genotype is an important predictor of resistance phenotype. However, this probably does not fully explain all the variance in the resistance phenotypes [17–19]. The substitution of a single Leu with Phe in the sodium channel was initially reported for *Musca domestica* (housefly) and was associated with a *kdr* mutation conferring pyrethroid resistance [20]. Subsequently, the *kdr* mutation has been identified from several other insect species [19, 21].

In wild-type individuals, the amino acid at position 1014 of *kdr* is Leu, whereas four mutation types (1014F, 1014S, 1014C and 1014W) have been detected in more than 13 *Anopheles* species in Africa, Asia and the Americas [19]. These *kdr* variants have been found to be most numerous in China [11, 22–25], among which the 1014F variant is the most prevalent throughout China [11], whereas the 1014C and 1014S variants are distributed mainly on the central and eastern coasts [11, 22, 23] and the southwest [24, 25] of China, respectively. To date, however, the 1014W variant has only been detected in Guangxi Province [25].

In the first decade of the present century, high resistance to pyrethroids and organochlorines was observed in populations of *Culex tritaeniorhynchus*, *Cx. pipiens pallens* and *Aedes albopictus* in nearly all surveyed districts of Shanghai [26, 27]. However, few surveys of the insecticide resistance status of these vectors of human pathogens have been reported in Shanghai in the past ten years. Moreover, the status of insecticide resistance in the primary malaria vector *An. sinensis* remains unknown, and there have been no analyses of target resistance gene mutations or their association with resistance phenotypes in Shanghai. Thus, concerted research effort is necessary to gain a better understanding of the insecticide susceptibility status in local *An. sinensis* populations in order to develop effective and sustainable insecticide resistance management strategies in this region. In our previous study, we found that *kdr* mutations were uniformly detected in all samples from the Songjiang, Jiading and Qingpu Districts of Shanghai, and from Xinhe County in the Chongming District. In contrast, we were unable to detect any *kdr* mutations in samples collected from the nearby Dongtan National Nature Reserve in Chongming District. Unfortunately, these samples were pooled for mosquito pathogen detection [28, 29]. Chongming District (also known as Chongming Island) is an island in the Yangtze estuary, separated from mainland Shanghai by the Yangtze River. The fact that the distance between Dongtan and Xinhe County in Chongming District is approximately 40 km could account for the marked difference in *kdr* mutation frequency between these two areas. In the present study, we assessed the levels of pyrethroid and organophosphate resistance in two *An. sinensis* populations from Xinhe and Dongtan, Chongming District, and examined distributions, polymorphism, genotype frequencies of target-site mutations in *VGSC* and the *ace-1* genes in Shanghai.

## Methods

### Mosquito sampling and bioassay preparation

**Fig. 1:**
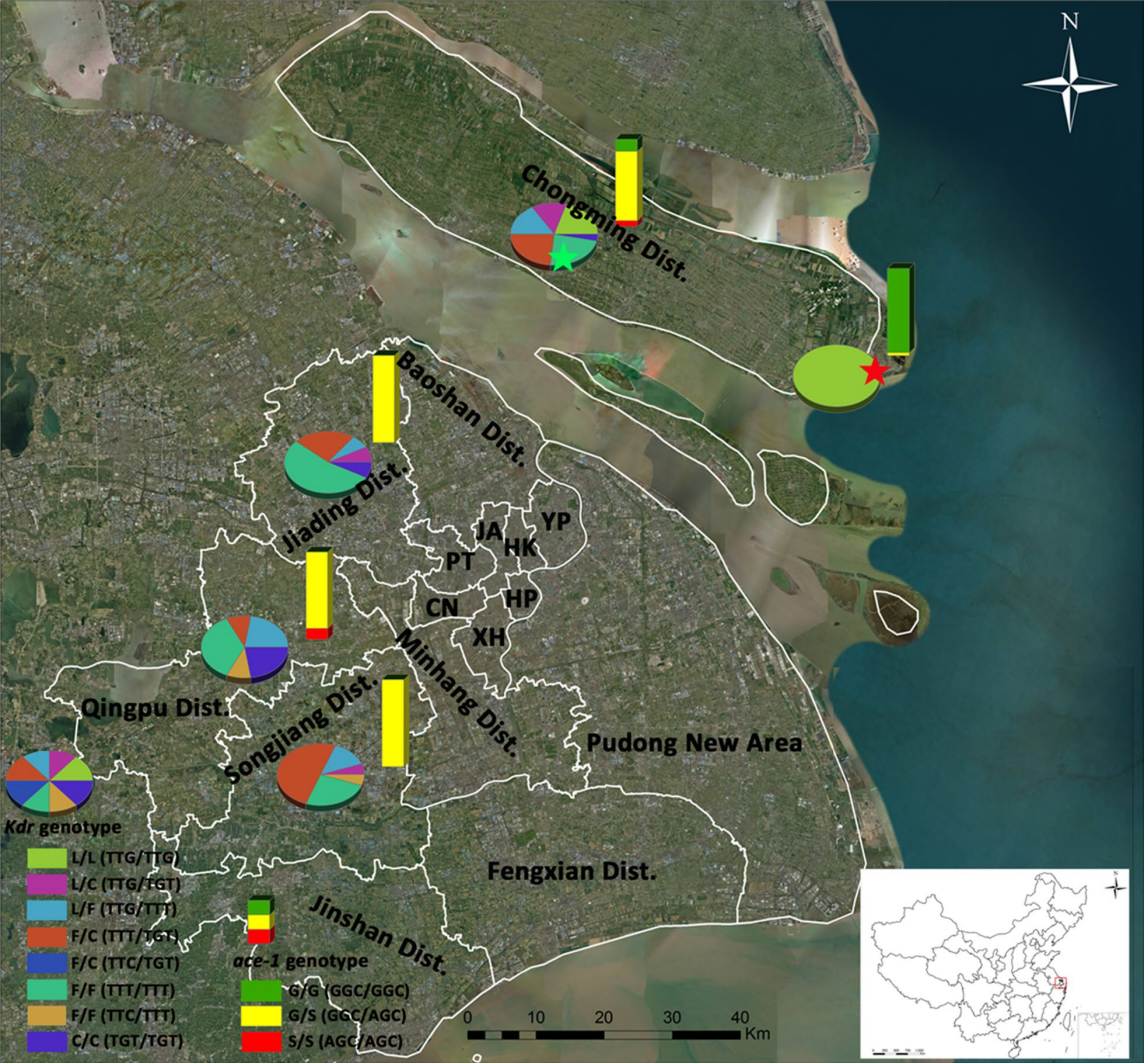
Map of the five sampling sites for *kdr* and *ace-1* genotype detection in Shanghai. Pie charts show the *kdr* genotypes and their frequencies. Histograms show the *ace-1* genotypes and their frequencies. Red and green stars represent the sampling sites for the CM-Dongtan and CM-Xinhe populations, respectively. *Abbreviations*: HP, Huangpu District; XH, Xuhui District; CN, Changning District; JA, Jing’an District; PT, Putuo District; HK, Hongkou District; YP, Yangpu District

Mosquito sampling for detection of the geographical distribution, polymorphism and mutation frequencies of insecticide resistance genes was based on routine mosquito surveillance in the Chongming, Songjiang, Qingpu and Jiading Districts of Shanghai (Fig. 1). Collections were made in the active mosquito season from July to October in 2016 and 2017, and we plotted a mosquito sampling map using ArcGIS ArcMap v.10.1 (ESRI, Redlands, CA, USA). The collection sites were mainly distributed in the sub-rural and rural regions where conditions are suitable for *An. sinensis* proliferation. The mosquito populations were named CM-Dongtan (near Dongtan National Nature Reserve, Chongming District), CM-Xinhe (Xinhe County, Chongming District), SJ (Songjiang District), JD (Jiading District) and QP (Qingpu District). Mosquitoes were identified using morphological characteristics according to the national key [30]. *Anopheles sinensis* adult mosquitoes were individually preserved in 2-ml centrifuge tubes and stored at − 20 °C for further molecular tests.

In order to determine the link between the target-site genotype of insecticide resistance genes and resistance phenotype, and characterize the insecticide resistance status of *An. sinensis* in the region, two field populations from two livestock farms near paddy fields in Chongming District were collected for this study during the period from August to October 2017. The two farms are separated by a distance of 40 km, one of which, located in Xinhe County in a rural area near the downtown district, is subjected to regular applications of insecticides for agriculture and public health purposes. The other farm is situated close to the sparsely populated Dongtan National Nature Reserve and promotes organic agriculture. To minimize the influence of mosquito age and blood-feeding history on resistance, and because larvae could not always be collected in appropriate numbers in the field, blood-fed female mosquitoes were caught individually using a transparent plastic tube containing moistened filter paper at the base. The collection tubes were brought back to the laboratory, and the blood-fed females were supplied with 10% sucrose to provide sufficient energy during the oviposition period. For the purposes of insecticide bioassays, we used 3- to 5-day-old F_1_ generation female mosquitoes. As a susceptible mosquito control, we used a laboratory-reared strain of *An. sinensis*, which has been maintained in the insectary of the Jiangsu Institute of Parasitic Diseases in Wuxi, China for 15 years in the absence of insecticide exposure. Temperature and humidity for mosquito rearing and the bioassay were controlled at 27 ± 1 °C and 70% ± 5%, respectively.

### Insecticide resistance bioassay

Resistance/susceptibility to pyrethroids (0.15% cyfluthrin, 0.05% α-cypermethrin and 0.05% deltamethrin) and an organophosphate (1% fenitrothion) was determined using the standard WHO resistance tube and protocol for adult mosquitoes [31]. Cyfluthrin, α-cypermethrin and fenitrothion are currently used for public health applications in Chongming District, whereas deltamethrin is widely used for against mosquitoes for public sanitation [10, 13, 32, 33]. Five to seven replicates of 20–25 individuals per tube were performed for assessing the resistance status of test mosquitoes against each insecticide. For the assays, the test mosquitoes were initially blown into a recovery tube where they were allowed to rest for 1 h before coming into contact with insecticide-impregnated paper, to which they were exposed for 60 min. The number of mosquitoes knocked down was recorded every 5 min for the first 20 min, and then at 10-min intervals for the remaining 40 min. After 1 h, the mosquitoes were maintained in the recovery tube for 24 h and supplied with fresh 10% sucrose solution prior to scoring mortality. A mosquito was classified as dead if immobile or unable to stand or fly in a coordinated manner. The mortality rate for each insecticide was adjusted according to the control mortality (> 5% but < 20%) based on Abbott’s formula [31]. After performing the resistance bioassay, the mosquitoes were placed in separate 2-ml centrifuge tubes and stored at − 20 °C until subsequent DNA extraction.

### DNA extraction and target gene mutation sequencing

For DNA extraction, samples were homogenized in a mixer mill (Jingxin, Shanghai, China) after 250 μl of ATL (Qiagen, Hilden, Germany), 20 μl of proteinase K (Qiagen) and two 3-mm steel balls were added to each tube. The mixture was incubated at 56 °C overnight in an oscillation thermo-block. The samples were then centrifuged at 1000 × *rpm* for 3 min at room temperature. Next, 200 μl of supernatant from each ground sample was added to the Roche® MagNA Pure 96 sample plate. The latter was performed using a MagNA Pure 96 System (Roche, Basel, Switzerland), as described in a previous study [29]. To detect *kdr* mutations, the region of the *VGSC* gene containing codon 1014 was PCR amplified using the primer pair *kdr*-F (5′-TGC CAC TCC GTG TGT TTA GA-3′) and *kdr*-R (5′-GAG CGA TGA TGA TCC GAA AT-3′) [23]. The *ace-1* gene was amplified using the primers 467F (5′-GTG CGA CCA TGT GGA ACC-3′) and 660R (5′-ACC ACG ATC ACG TTC TCC TC-3′) [22], which generated a 193-bp PCR fragment encompassing target codon 119. The amplification products were separated by agarose gel electrophoresis, purified, and sequenced in both directions by Sangon Biotech (Shanghai, China). The peak spectrum of the sequencing data was determined using Bio-edit v.7.2.6 [34]. To confirm the allelic information for certain ambiguous sequences, cloning and plasmid sequencing were performed.

### Statistical analyses

The time required for 50% knockdown (KT_50_) of the mosquitoes was determined using a log-time probit model. To compare the status of insecticide resistance, differences in mortality rates among different *An. sinensis* populations were analysed by univariate analysis of variance (ANOVA) using the *arcsin*-transformed data of mosquito mortality rate. Associations between each *kdr* or *ace-1* genotype and phenotypical resistance/susceptibility were evaluated by logistic regression. All analyses were conducted using SPSS v.20 (IBM Corp., Armonk, NY, USA).

## Results

### Insecticide susceptibility bioassay

We examined resistance to pyrethroid and organophosphate insecticides in two mosquito populations (CM-Dongtan and CM-Xinhe). The insecticide bioassay was performed using a susceptible laboratory strain and two field populations from Chongming District, the CM-Dongtan and CM-Xinhe populations. The mortality rates and KT_50_ values of these populations with respect to cyfluthrin, α-cypermethrin, deltamethrin and fenitrothion are shown in Table 1. For all test insecticides, the mortality rates of the sensitive reference strain were > 98%. Although the CM-Dongtan population was sensitive to deltamethrin and fenitrothion, we detected probable resistance to cyfluthrin (mortality: 91.90%) and α-cypermethrin (mortality: 81.19%). However, ANOVA indicated no significant differences between the reference strain and the CM-Dongtan population in term of mortality rates for the four tested insecticides. Conversely, we found that the CM-Xinhe population was highly resistant to all tested insecticides, with the mortality rate in each case being < 80%, which were significantly lower compared with the laboratory reference strain. Specifically, CM-Xinhe samples were highly resistant to α-cypermethrin and deltamethrin, with mortality rates of 21.23% and 26.57%, respectively, whereas they were moderately resistant to cyfluthrin (mortality: 64.76%) and fenitrothion (mortality: 51.77%).

**Table 1:** Pyrethroid and organophosphate resistance of a laboratory susceptible strain and F1 progeny of two field populations of *Anopheles sinensis* from Chongming District, Shanghai

Population	0.15% cyfluthrin				0.05% α-cypermethrin				0.05% deltamethrin				1% fenitrothion			
	*n*	KT_50_/min (95% CI)	Mort (%) (mean ± SD)	Status	*n*	KT_50_/min (95% CI)	Mort (%) (mean ± SD)	Status	*n*	KT_50_/min (95% CI)	Mort (%) (mean ± SD)	Status	*n*	KT_50_/min (95% CI)	Mort (%) (mean ± SD)	Status
Laboratory susceptible strain	120	10.61 (9.22–11.76)	100.00 ± 0.00	S	123	21.18 (19.60–22.86)	99.02 ± 0.58	S	121	17.19 (16.09–18.32)	100.00 ± 0.00	S	132	55.05 (50.73–61.15)	98.61 ± 1.76	S
CM-Dongtan population	105	16.69 (15.15–18.22)	91.90 ± 7.33	PR	144	20.46 (19.10–21.93)	81.19 ± 12.19	PR	120	24.71 (22.34–27.20)	98.03 ± 3.40	S	123	109.50 (76.71–558.23)	100.00 ± 0.00	S
CM-Xinhe population	174	64.72 (52.22–98.61)	64.76 ± 6.17^**^	R	150	77.29 (60.87–114.90)	21.23 ± 7.02^**^	R	108	89.56 (65.77–162.03)	26.57 ± 20.01^**^	R	108	130.14 (86.65–506.19)	51.77 ± 16.11^*^	R

*Abbreviations*: KT_50_, 50% knockdown resistance; Mort, mortality rate; CM, Chongming; CI, confidence interval; S, susceptible (mortality rate > 98%); SD, standard deviation; PR, probably resistant (mortality rate 80–98%); R, resistant (mortality rate < 80%)

*P < 0.05; **P < 0.01

The resistance character of the CM-Xinhe population was also reflected in the KT_50_ values obtained from the insecticide susceptibility bioassays. KT_50_ values determined for the susceptible strain and CM-Dongtan population were in the ranges of 10.61–21.18 min and 16.69–24.71 min, respectively, for the three pyrethroids and 55.05 and 109.50 min, respectively, for fenitrothion. In contrast, for the CM-Xinhe population, the KT_50_ values determined for cyfluthrin, α-cypermethrin, deltamethrin and fenitrothion were 64.72, 77.29, 89.56 and 130.14 min, respectively, which represent fold increases of between 2.36 and 6.10 compared with the values obtained for the reference laboratory strain.

### *kdr* and *ace-1* allele frequencies in the *An. sinensis* populations

**Fig. 2:**
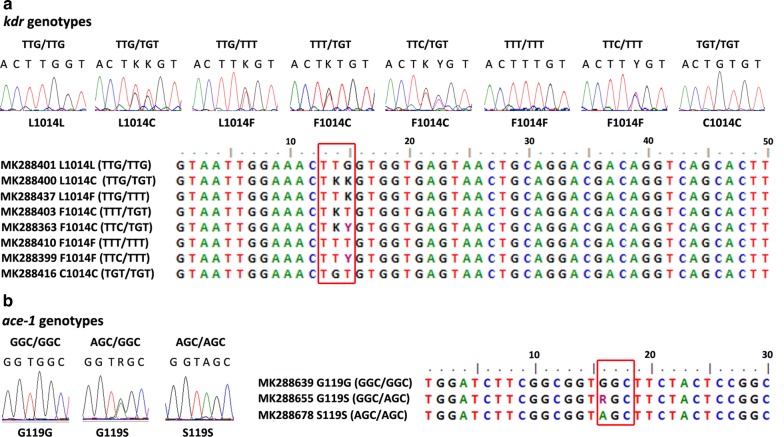
Chromatograms and alignments of the *kdr* and *ace-1* genotypes detected in *Anopheles sinensis* from Shanghai. **a** Eight *kdr* genotypes; codon 1014 of the *para*-type voltage-gated sodium channel gene is indicated by a red box. **b** Three *ace-1* genotypes; codon 119 of the *ace-1* gene is indicated by a red box. Different peak colours distinguish the four bases T (red), C (blue), A (green) and G (black). K = G/T; Y = T/C; R = A/G

We genotyped 103 dead (susceptible), 42 live (resistant) and 34 susceptible laboratory-bred mosquitoes after bioassays, together with 93 other mosquitoes collected from routine surveillance (GenBank accession numbers of *kdr* genotypes: MK288196–MK288467; *ace-1* genotypes: MK288468–MK288736). The samples used for detection of target gene mutation consisted of six populations: one susceptible laboratory strain and five field populations (CM-Dongtan, CM-Xinhe, SJ, JD and QP). We accordingly detected three types of mutant *kdr* alleles, two of which (TTT and TTC) resulted in a change from Leu to Phe, whereas the third (TGT) caused a Leu to Cys substitution (Table 2). Details of the geographical distributions, polymorphisms and frequencies of *kdr* and the *ace-1* genotypes for each of the five field sampling sites are shown in Fig. 1. Eight *kdr* genotypes were detected in the *An. sinensis* populations of Shanghai (Fig. 2a). The susceptible *kdr* genotype TTG/TTG was observed only in populations from the Chongming District. The TTG allele was detected at a moderate level (34.40%) in the CM-Xinhe population but only at low frequencies in the SJ, JD and QP populations (9.09%, 6.67% and 11.11%, respectively). With the exception of the Dongtan population, TTT was the dominant allele in all wild populations with a frequency ranging between 42.66–70.00%. TTC was comparatively rare, with only a single allele detected for each of the CM-Xinhe, SJ and QP populations. All TTC mutations were heterozygous, being found in combination with either TTG or TTT. The *kdr* genotype in the CM-Xinhe population was the most diverse in terms of polymorphism and included TTG/TTG, TTG/TGT, TTG/TTT, TTT/TGT, TTT/TTT, TTC/TGT and TGT/TGT genotypes. The frequencies of the first five genotypes ranged between 14.68–23.85%, whereas frequencies of the latter two were 0.92% and 2.75%, respectively. TTC/TGT was unique to the CM-Xinhe population, whereas TTC/TTT could not be detected in this population. The TTC/TTT genotype was, however, detected in the SJ and QP populations. The *kdr* genotype TTT/TTT was dominant in the Jiading and Qingpu Districts, and the TTT/TGT genotype was the primary *kdr* genotype in the Songjiang District.

**Table 2:** Distributions, polymorphisms and mutation frequencies of *kdr* alleles and genotypes in *Anopheles sinensis* populations in Shanghai

Population	CM-Dongtan population		CM-Xinhe population		Songjiang population		Jiading population		Qingpu population	
Sample size	*n*	Frequency (%)	*n*	Frequency (%)	*n*	Frequency (%)	*n*	Frequency (%)	*n*	Frequency (%)
*kdr* allele										
TTG (L)	234	100	75	34.40	4	9.09	2	6.67	2	11.11
TTT (F)			93	42.66	27	61.36	21	70.00	10	55.56
TGT (C)			49	22.48	12	27.27	7	23.33	5	27.78
TTC (F)			1	0.46	1	2.27			1	5.56
*kdr* genotype										
L/L (TTG/TTG)	117	100	21	19.27						
L/C (TTG/TGT)			17	15.60	1	4.55	1	6.67		
L/F (TTG/TTT)			16	14.68	3	13.64	1	6.67	2	22.22
F/C (TTT/TGT)			25	22.94	11	50.00	4	26.67	1	11.11
F/C (TTC/TGT)			1	0.92						
F/F (TTT/TTT)			26	23.85	6	27.27	8	53.33	3	33.33
F/F (TTC/TTT)					1	4.55			1	11.11
C/C (TGT/TGT)			3	2.75			1	6.67	2	22.22

*Abbreviations*: *kdr*, knockdown resistance; CM, Chongming District; *n*, number; L, Leu; F, Phe; C, Cys

Three *ace-1* genotypes were detected in the surveyed *An. sinensis* populations (Fig. 2b). The *ace-1* mutant allele AGC was detected in all five field populations, and had a frequency of 1.71% in the CM-Dongtan population (Table 3). All CM-Dongtan samples characterized by the AGC/GGC genotype were collected during the mosquito surveillance of 2016. In contrast, no mutations were detected in any of the samples collected in 2017. The GGC and AGC alleles were evenly distributed at frequencies of between 43.75 and 56.25% in the *An. sinensis* populations, except the CM-Dongtan population. The heterozygote GGC/AGC was the dominant *ace-1* genotype in the CM-Xinhe (79.44%), SJ (100.00%), JD (100.00%) and QP (87.50%) populations. The susceptible GGC/GGC genotype was confined to the Chongming District, whereas the homozygote AGC/AGC genotype was detected in the CM-Xinhe and QP populations at frequencies of 6.54% and 12.50%, respectively.

**Table 3:** Distributions, polymorphisms and mutation frequencies of *ace-1* alleles and genotypes in *Anopheles sinensis* populations in Shanghai

Population	CM-Dongtan population		CM-Xinhe population		Songjiang population		Jiading population		Qingpu population	
Sample size	*n*	Frequency (%)	*n*	Frequency (%)	*n*	Frequency (%)	*n*	Frequency (%)	*n*	Frequency (%)
*ace-1* allele										
GGC (G)	230	98.29	114	53.27	22	50.00	15	50.00	7	43.75
AGC (S)	4	1.71	100	46.72	22	50.00	15	50.00	9	56.25
*ace-1* genotype										
G/G (GGC/GGC)	113	96.58	15	14.02						
G/S (GGC/AGC)	4	3.42	85	79.44	22	100	15	100	7	87.50
S/S (AGC/AGC)			7	6.54					1	12.50

*Abbreviations*: *ace-1*, acetylcholinesterase 1; CM, Chongming District; *n*, number; G, Gly; S, Ser

### Relationship between target gene mutation and phenotypic resistance

Subsequent to performing the insecticide bioassay, we randomly genotyped the partial *VGSC* and *ace-1* genes of both surviving and dead individuals (Tables 4, 5). The genotypes of all the samples from CM-Dongtan population after bioassays were susceptible. Thus, the relationship between target gene mutation and phenotypic resistance was analysed in the CM-Xinhe population. We accordingly detected a significant association (*P* = 0.005) between the homozygote 1014F/1014F genotype and resistance to the pyrethroids, with an odds ratio of 0.133 (95% confidence interval, CI: 0.033–0.536). Furthermore, we found that the homozygous resistant type 1014F/1014F was significantly linked (*P* = 0.024) to cyfluthrin resistance. However, no significant association was detected between the other *kdr* or *ace-1* genotypes and phenotypic resistance to the tested insecticides.

**Table 4:** Frequencies of *kdr* genotypes in relation to mosquito phenotypical resistance/susceptibility as determined by resistibility bioassays of pyrethroid and organophosphate in F1 progeny of two *Anopheles sinensis* populations from Chongming District, Shanghai

Population	Insecticide	Status	*n*	Frequencies of *kdr* genotypes (%)					
				1014L/1014L	1014L/1014F	1014L/1014C	1014F/1014C	1014C/1014C	1014F/1014F
CM-Xinhe population	0.15% cyfluthrin	Alive	8	12.50			25.00		62.50*
		Dead	15	13.33	13.33	26.67	33.33		13.33
	0.05% α-cypermethrin	Alive	12	8.33	16.67	8.33	33.33		33.33
		Dead	9	33.33	11.11	33.33	22.22		
	0.05% deltamethrin	Alive	9	11.11	11.11		33.33	11.11	33.33
		Dead	11	27.27	27.27	9.09	18.18	9.09	9.09
	1% fenitrothion	Alive	8		12.50		12.50		75.00
		Dead	7	14.28	14.28	14.28	28.57		28.57
CM-Dongtan population	0.15% cyfluthrin	Alive	1	100					
		Dead	21	100					
	0.05% α-cypermethrin	Alive	4	100					
		Dead	17	100					
	0.05% deltamethrin	Alive	1	100					
		Dead	23	100					
	1% fenitrothion	Alive	–						
		Dead	22	100					

*Abbreviations*: *kdr*, knockdown resistance; CM, Chongming District; *n*, number; L, Leu; F, Phe; C, Cys

*P < 0.05

**Table 5:** Frequencies of *ace-1* genotypes in relation to mosquito phenotypical resistance/susceptibility as determined by resistibility bioassays of pyrethroid and organophosphate in F1 progeny of two *Anopheles sinensis* populations from Chongming District, Shanghai

Population	Insecticide	Status	*n*	Frequencies of *ace-1* genotypes (%)		
				119G/119G	119G/119S	119S/119S
CM-Xinhe population	0.15% cyfluthrin	Alive	7	14.29	85.71	
		Dead	14	14.28	78.57	7.14
	0.05% α-cypermethrin	Alive	12	8.33	91.67	
		Dead	9	22.22	77.78	
	0.05% deltamethrin	Alive	9		100	
		Dead	10	10.00	60.00	30.00
	1% fenitrothion	Alive	8		100	
		Dead	7	14.29	85.71	
CM-Dongtan population	0.15% cyfluthrin	Alive	1	100		
		Dead	21	100		
	0.05% α-cypermethrin	Alive	4	100		
		Dead	17	100		
	0.05% deltamethrin	Alive	1	100		
		Dead	23	100		
	1% fenitrothion	Alive	–			
		Dead	22	100		

*Abbreviations*: *ace-1*, acetylcholinesterase 1; CM, Chongming District; *n*, number; G, Gly; S, Ser

## Discussion

### Phenotypic resistance status in Chinese populations of *An. sinensis*

Comparisons of the KT_50_ values and mortality rates among the susceptible laboratory strain and the CM-Dongtan and CM-Xinhe populations revealed that the Dongtan population is potentially susceptible to those insecticides tested, including cyfluthrin and α-cypermethrin. This finding was supported by the target-site genotyping analyses. In contrast, the CM-Xinhe population, some ~ 40 km distant from Dongtan on the same island, is resistant to pyrethroids and an organophosphate. We found that the resistance status of various *An. sinensis* populations differ significantly from one another. The sampling sites at Dongtan are located near a National Nature Reserve and characterized by wetland established by enclosure of the land by the sea. Given its proximity to the nature reserve, this area has benefited from the promotion of organic farming, along with the strict control of pesticide usage. In contrast, the patterns of insecticide usage in CM-Xinhe County are comparable to those of mainland Shanghai. Indeed, both CM-Xinhe County and mainland Shanghai have had a long history of insecticide application for the control of agricultural pests and protection of public health.

Within China, the Yangtze valley region, which encompasses the provinces of Jiangsu, Zhejiang, Anhui, Jiangxi, Hunan, Hubei, Guizhou and Sichuan, Shanghai City and the northern parts of Shanxi provinces, accounts for two-thirds of the total national rice planting area and yield. The annual double-rice cropping cultivation practiced in this region is associated with extensive insecticide use and multiple sprays during each growing season, in order to prevent losses of rice yield and quality from severe insect pest damage. Given that rice paddies are a major breeding ground for *An. sinensis* larvae, a corollary of this practice is that *An. sinensis* larvae are subjected to a constant and intensive insecticide selection pressure. Available data indicates the current high resistance of *An. sinensis* to deltamethrin in China, with bioassay mortality rates of 9.8–15.1%, 26.6–54.3% and 29.6–67.4% being recorded in populations from central China (dominant double-rice product area, Hunan and Hubei provinces [23, 32]), eastern China (secondary double-rice product area, Anhui Province [23, 35] and Shanghai) and southwestern China (single-rice area, Yunnan Province [32, 35]), respectively. These data indicate that the tendency of pyrethroid resistance levels may be correlated with rice yields in mainland China. It is conceivable that the migration of mosquitoes between untreated and treated areas may delay the development of insecticide resistance by diluting the indigenous resistant populations with susceptible migrants [32, 36, 37]. In this regard, we might predict that, owing to the influences of wind and international trade, there would be a more frequent migration of mosquitoes to Anhui Province and Shanghai City, which are located near the eastern coast of China, than to central China, where migration could be impeded by north-south mountain ranges. This latter physical barrier to mosquito migration could partially explain why the insecticide resistance status is more severe in central than eastern China. In Yunnan Province, *An. sinensis* populations are characterized by a phenotypically lower insecticide resistance and no *kdr* mutations [22], which could be attributable to the predominant practice of single-rice cropping in this province. Moreover, Yunnan lies in a region of China bordering Laos, Myanmar and Vietnam, where agricultural methods are less developed than those in China and pesticide usage is typically low. Although *kdr* mutations have been detected in some regions of southern Vietnam and Cambodia, these tend to be located at some distance from Yunnan Province.

### Distribution, genotypes and frequencies of *kdr* and *ace-1* mutations in the *An. sinensis* populations of Shanghai

The high insecticide resistance detected in the CM-Xinhe population of *An. sinensis* is reflected in the 65.60% frequency of *kdr* mutations in this population. In contrast, we detected no *kdr* mutations among any of the 117 specimens collected from the CM-Dongtan population. Whereas the frequency of the *kdr* susceptible homozygote TTG/TTG genotype detected in the Xinhe population was 19.27%, none of the three populations surveyed from mainland Shanghai carried this genotype. Therefore, we speculate that the *kdr* mutant allele is probably stable in the SJ, QP and JD populations. The prevalence of resistance genes in mainland Shanghai is primarily a consequence of long-term agricultural pesticide use, aggravated by public health-related insecticide application to control vector-borne diseases. In addition, the Yangtze River separates Chongming Island and mainland Shanghai, and represents a geographical boundary that hinders mosquito population migration between the two regions.

We identified two amino acid replacements at codon 1014 of the VGSC protein, namely 1014F and 1014C, which are common in Central and East Asia [19]. Two other substitutions reported in China, 1014S and 1014W, have been identified in Guangxi Province [25], the former of which is detected mainly in Southwest Asia. In China, it has also been detected in Guangdong Province [38], and in other members of the Hyrcanus Group in the Greater Mekong Subregion, including Vietnam, Cambodia and Laos [39]. 1014W was detected in samples from Guangxi Province in 2012 [25], but to date has yet to be found elsewhere. The occurrence of 1014C in *An. sinensis* was first reported in Korea [36] and subsequently in the Chinese provinces of Anhui [23], Hunan, Hubei, Jiangsu [32], Sichuan [11], Guizhou and Guangxi [24]. Nevertheless, it has yet to be detected in Guangdong Province in China or in the *An. sinensis* populations of other Southeast Asian countries. The 90.91%, 93.33% and 88.89% *kdr* mutation frequencies we detected in the SJ, JD and QP populations, respectively, are comparable with those reported for Anhui, Henan, Jiangsu and Hubei provinces, wherein *kdr* mutation frequencies were all greater than 90% [38]. *kdr* mutations appear to have stabilized in the *An. sinensis* populations in these regions, and susceptible *An. sinensis* strains with no resistance gene mutation are seldom encountered in the central or eastern regions of China. The prevalence of a *kdr* susceptible genotype in the CM-Dongtan population is probably the result of the National Reserve Programme, which aims to protect the area from the wide use of insecticides. The rates of *kdr* mutation previously reported in northeastern and the southeastern Guangxi Province were 50.0% and 11.6–33.3%, respectively [24], and have generally been found to be less than 35% in Guizhou, Guangdong, Fujian and Shandong [38]. In addition to an association with the trend of the pyrethroid resistance distribution in China, the frequency of *kdr* mutation may also be linked to rice yields.

The influence of geographical boundaries on gene flow in *An. sinensis* populations is also reflected in the genotypic distribution of the *ace-1* gene. We found that the susceptible genotype of *ace-1* is confined to the Chongming District and that nearly all Dongtan samples were characterized by the GGC/GGC *ace-1* genotype. However, four heterozygote GGC/AGC genotypes were detected in the samples collected from this area in 2016. The GGC/AGC genotype comprised 79.44% of the samples from the CM-Xinhe population, and thus gene flow may have occurred between the two populations. It was reported that the frequencies of *ace-1* 119S mutations in the Yunnan and Anhui populations were moderate, whereas frequencies of *kdr* mutation in these population show considerable variation [22, 38]. In the present study, all examined samples from the Songjiang and Jiading Districts had a GGC/AGC genotype. With the exception of the Dongtan population, the frequency of this genotype was 84.86% in the other four populations from Shanghai, which is higher than that detected in the populations inhabiting Anhui, Guangxi, and Yunnan provinces (63.57%, 33.01% and 46.11%, respectively) [40]. We found that the frequency of the homozygote AGC/AGC genotype was comparatively low in Shanghai, and was detected only in the CM-Xinhe and QP populations at frequencies of 6.54% and 12.50%, respectively. In Guangxi Province, frequencies of the homozygote mutant genotypes of *ace-1* ranged between 33.3–85.7% [40].

In contrast to the frequency of *kdr* mutation, which is higher in central and eastern China than in southern and southwestern China, we found that the *ace-1* homozygote mutation frequency is higher in southwestern China than in central and eastern China, which may reflect the type and quantity of insecticide used in these two regions.

### Positive association between the homozygote 1014F/1014F genotype and the resistance phenotype

Although there have been some doubts as to the relationship between *kdr* mutations and phenotypic resistance [39, 41], a positive correlation between these two factors has been established [19]. In the present study, we detected a significant correlation between the homozygote 1014F/1014F genotype and pyrethroid resistance in the CM-Xinhe population. In contrast, we observed no obvious associations between heterozygote or other homozygote mutation genotypes and phenotypic resistance. This tends to indicate that the *kdr* mutations associated with insecticide resistance are probably recessive, which is consistent with the speculations of Donnelly et al. [18]. We speculate that the combination of 1014L and 1014C mutations may weaken the influence of the 1014F mutation on the resistance trait. However, further studies are needed that increase the sample size to conclusively determine the role of the *kdr* 1014C allele with its heterozygote and homozygote genotypes on insecticide resistances.

Mutation of the *ace-1* gene has previously been shown to be correlated with organophosphate and carbamate resistance [42]. Furthermore, a positive association between the 119S mutation and malathion resistance has been identified in *An. sinensis* populations from Anhui and Yunnan provinces [22], whereas in contrast, no significant relationship was found between *ace-1* mutation and fenitrothion resistance in this study.

### Limitations of this study

One limitation of the present study was the small number of samples for detection of target gene mutation after bioassays. In order to gain a more comprehensive understanding of the associations between target gene mutations and resistance phenotypes, and to elucidate the mechanisms underlying insecticide resistance in the *An. sinensis* populations of Shanghai, it will be necessary to obtain a larger number of samples for sequencing insecticide resistance genes.

Modern-day Shanghai is an international metropolis, with the distribution of croplands limited in sub-rural and rural areas, and in recent years, *An. sinensis* has become scarce in the central area of the city. Accordingly, samples used in the present study to examine the geographical distribution and polymorphism of target-site mutations in the *VGSC* and the *ace-1* gene were, of necessity, obtained from sub-rural and rural districts. However, the collection sites used for routine mosquito surveillance do not cover all districts with a sizeable *An. sinensis* population. Consequently, comprehensive coverage and systematic sampling are needed for further examinations of the geographical distribution and polymorphism of insecticide resistance genes in the *An. sinensis* populations of Shanghai.

## Conclusions

In the present study, we detected high frequencies of *kdr* and *ace-1* mutations in *An. sinensis* populations, particularly those inhabiting mainland Shanghai. Thus, continued use of the insecticides currently being applied to control *An. sinensis* in these areas may aggravate the resistance problem by diluting the frequency of susceptible alleles in the population. Similarly, reducing insecticide usage in the field through rational management and the promotion of organic agriculture may lower the frequency of target gene mutations, since the mutant alleles of *kdr* and *ace-1* may have certain fitness costs in the absence of insecticide [43, 44]. In addition, the results of our bioassay of the *An. sinensis* population inhabiting the central Chongming District of Shanghai can be regarded as indicative of the high insecticide resistance status of the *An. sinensis* populations in the city. These findings highlight the importance of longitudinal insecticide resistance monitoring and the urgent need to determine the current status of insecticide resistance in this region. Continued monitoring of insecticide resistance and molecular studies focussing on the genotypic diversity of resistance genes may assist in predicting potential changes in the strength of resistance to pesticides applied for public health purposes.

## Data Availability

All data generated or analysed during this study are included in this published article. The newly generated sequences were submitted to the GenBank database under the accession numbers MK288196–MK288736.

## Abbreviations

*ace-1*: acetylcholinesterase 1
ANOVA: univariate analysis of variance
CM: Chongming
JD: Jiading
*kdr*: knockdown resistance
KT_50_: 50% knockdown
QP: Qingpu
SJ: Songjiang
VGSC: voltage-gated sodium channel
WHO: World Health Organization
